# Necrotizing Pseudomonal Sinusitis in a Transplant Patient

**DOI:** 10.7759/cureus.93116

**Published:** 2025-09-24

**Authors:** Charlyn N Gomez, Manaahil Rao, Katya Prakash, Andrea M Hebert, Sunny J Haft

**Affiliations:** 1 Department of Otorhinolaryngology - Head and Neck Surgery, University of Maryland School of Medicine, Baltimore, USA; 2 Department of Radiation Oncology, University of Maryland School of Medicine, Baltimore, USA; 3 Department of Infectious Disease, University of Maryland School of Medicine, Baltimore, USA

**Keywords:** complicated sinusitis, invasive fungal sinusitis, necrotizing pseudomonal sinusitis, pseudomonas infections, transplant complication

## Abstract

Necrotizing pseudomonal sinusitis is a rare disease that can be clinically identical to invasive fungal sinusitis (IFS). Early differentiation between these two etiologies is crucial, as the treatment algorithms are distinct. Here, we present a report of necrotizing pseudomonal sinusitis in a solid organ transplant patient and propose an appropriate treatment strategy for this disease. A 65-year-old lung transplant patient developed a *Pseudomonas *bacteremia, followed by symptoms of a severe, complicated sinusitis that included a trigeminal neuropathy. Imaging showed extension of disease outside the sinonasal cavities, involving the periorbital and periantral spaces. The patient was treated with emergent surgical debridement confined to the sinonasal cavities. This was followed by long-term, focused systemic and topical antipseudomonal therapy, as well as modification of the patient's immunosuppressive regimen, resulting in full resolution of the patient's pseudomonal infection. Otolaryngologists, as well as transplant practitioners, should be aware of this rare, potentially fatal disease that so closely mimics IFS.

## Introduction

Sinonasal symptoms in immunocompromised patients can be due to a broad range of pathologies, ranging from recalcitrant bacterial infection to invasive fungal disease. Invasive fungal sinusitis (IFS) is a feared diagnosis within the transplant population, which can rapidly spread to surrounding neurovascular structures and cause significant morbidity and mortality [1]. This type of sinusitis carries a mortality rate anywhere from 50-80%, even with prompt surgical management [1]. The prevalence of IFS can overshadow a much more rare, but equally destructive and rapidly progressive form of sinusitis caused by *Pseudomonas aeruginosa *(*P. aeruginosa*). Transplant patients are at increased risk for developing necrotizing pseudomonal sinusitis due to impaired host defense mechanisms, including neutrophil function, macrophage phagocytosis, and mucosal immunity, which are critical for controlling *P. aeruginosa* and preventing tissue invasion and necrosis [2,3]. Necrotizing pseudomonal sinusitis is difficult to recognize because it so closely mimics that of invasive fungal sinusitis [4]. Acute and chronic sinusitis caused by *P.aeruginosa* is not uncommon, but only a handful of these cases report rapidly progressive necrotizing infection with local tissue destruction and avascular necrosis [4,5]. Other reported complications have included blindness, carotid artery thrombosis, cerebral abscess, and death [5]. The survival of patients afflicted with necrotizing pseudomonal sinusitis is critically dependent on early recognition, differentiation, and targeted treatment of this pathology. Mortality rates for initial infection episodes are reported to be approximately 38% in solid transplant patients [6]. To our knowledge, there have been no reported cases of invasive necrotizing *Pseudomonas *sinusitis in the solid organ transplant population.

In other words, most physicians treating transplant patients may initially consider invasive fungal infections when new sinus symptoms appear, as those are common and dangerous. However, we describe a different infection caused by *P. aeruginosa*, which can mimic fungal disease but requires a different treatment strategy. Recognizing this possibility in a timely fashion is essential to preventing treatment delays and lowering the risk of serious complications.

## Case presentation

**Figure 1:**
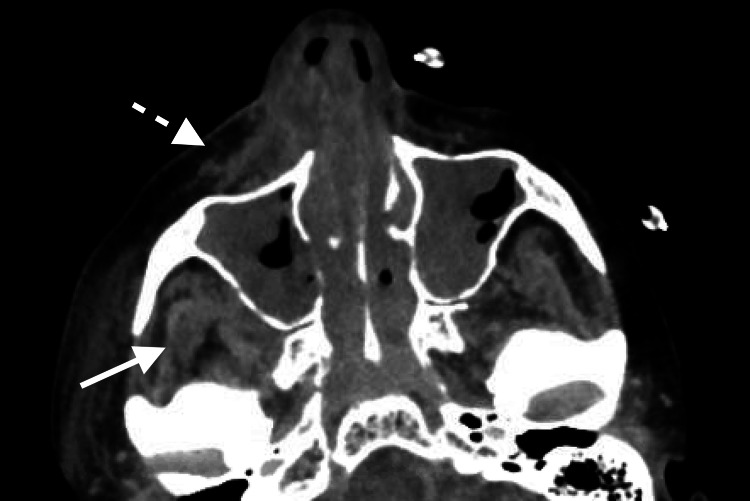
Pre-operative axial CT sinus After presenting with bilateral facial swelling, right maxillary tenderness, and V2 numbness, the patient was found to have fat stranding, thickening, and enhancement of the retroantral (thick arrow) and premaxillary (dashed arrow) spaces on the right, indicative of pseudomonal spread outside of the sinonasal cavity.

**Figure 2:**
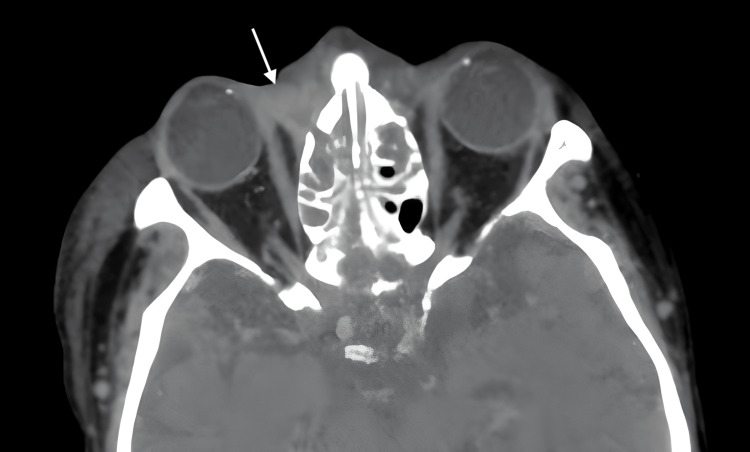
Pre-operative axial CT of periorbital space Preseptal enhancement within the periorbital space (white arrow) was also observed on imaging shortly after admission.

**Figure 3:**
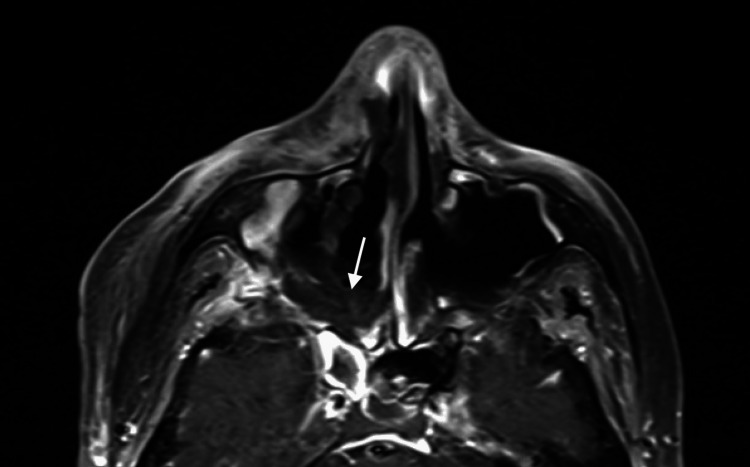
Post-operative day nine axial MRI sinus Paranasal sinus necrosis (white arrow) and multiple phlegmons were seen on imaging, resulting in the patient returning to the OR for repeat debridement.

**Figure 4:**
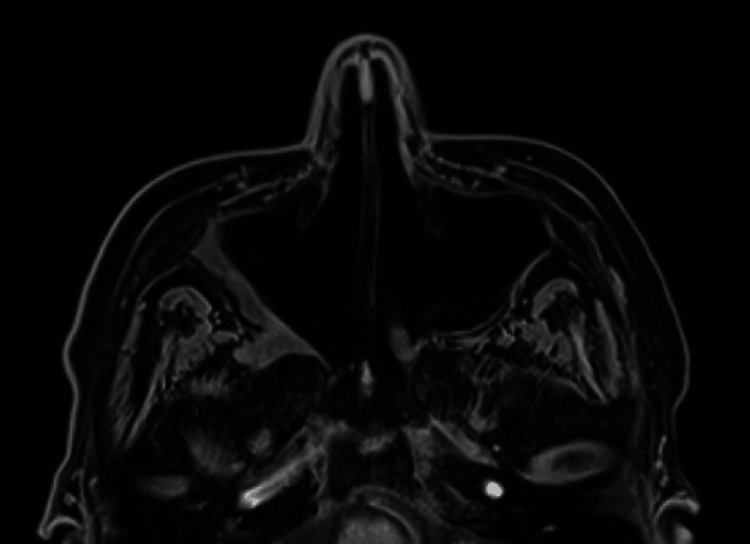
One-year post-operative MRI sinus No findings indicating invasive disease in the sinuses, indicating resolution of infection.

A 65-year-old man with a history of type II diabetes and idiopathic pulmonary fibrosis who underwent bilateral lung transplantation 18 months prior to presentation was admitted to the hospital with pseudomonal bacteremia. The patient had received basiliximab induction and was on maintenance immunosuppression with tacrolimus, prednisone, and azathioprine at the time of presentation. Shortly after admission, he developed bilateral facial swelling and right maxillary tenderness, along with trigeminal (V2) numbness. CT imaging showed pansinusitis, along with inflammation and stranding of the retroantral fat (Figure 1) and periorbital tissue (Figure 2), as well as thinning of the fovea ethmoidalis with associated dural enhancement. Nasal endoscopy performed at bedside was notable for an insensate right middle turbinate with pale mucosa, along with frankly necrotic mucosa of the bilateral inferior turbinates. Based on a CT sinus without contrast, which demonstrated pansinusitis with bony erosion involving the anterior, medial, and posterior maxillary sinus walls on the right, concerning for invasive fungal sinusitis, he was taken to the operating room emergently. Intraoperative frozen pathology revealed extensive necrosis, but the fungal culture was negative. No potassium hydroxide (KOH) samples were taken and evaluated. Given the absence of fungal angioinvasion, surgical resection was conservative, carried up to, but not past, the lamina papyracea or other bony limits of the sinonasal cavity. Over the subsequent days, abundant *P. aeruginosa* grew from intraoperative cultures. Cefepime, levofloxacin, and twice-daily tobramycin nasal irrigations were initiated. On post-operative day nine, he underwent an MRI, which showed persistent necrosis and multiple phlegmons within the paranasal sinus mucosa (Figure 3). The patient was taken back to the OR for additional sinus debridement. Sinus symptoms gradually abated over a 10-week course of the intravenous and topical antibiotics, but then recurred six months later. An updated MRI showed mucosal thickening and necrosis of bilateral sphenoid sinuses, as well as soft tissue enhancement along the right pterygopalatine fossa with extension into the pterygomaxillary fissure. Further OR debridement was not pursued, and focus was redirected to aggressive medical management. Immunosuppression on discharge included cyclosporine and prednisone, in addition to prophylactic atovaquone and valganciclovir. The patient was transitioned to cyclosporine from tacrolimus due to his altered mental status. Additional medical management included a combination of intravenous, oral, and intranasal anti-pseudomonal therapy, which was continued for eight months. One year following discontinuation of his antibiotic regimen, his sinonasal symptoms have fully resolved, and the patient has had no evidence of recurrent infection on endoscopy or MRI (Figure 4). The patient's vision has remained fully intact throughout his course, while V2 paresthesia has persisted. His clinical course is summarized in Table 1.

**Table 1:** Summary of the patient's clinical course Different time points, events, corresponding findings, and associated interventions with their respective outcomes are displayed. OR - operating room

Time point	Event	Findings	Intervention	Outcome
Admission	Hospitalized for pseudomonal bacteremia, 18 months post-lung transplant	Bilateral facial swelling, right maxillary tenderness, V2 numbness	Started inpatient management	Concern for invasive fungal sinusitis
Day 0	CT sinus + nasal endoscopy	Pansinusitis, retroantral/periorbital inflammation, thinning of fovea ethmoidalis, necrotic turbinates	Emergent OR for sinus surgery	Frozen section: necrosis, no fungal invasion
Days 1-8	Intra-operative cultures growing	Pseudomonas aeruginosa on bacterial culture	Started cefepime, levofloxacin, & tobramycin irrigations	-
Day 9	MRI sinus	Persistent necrosis, phlegmons in paranasal sinuses	Return to OR for repeat debridement	Continued IV + topical antibiotics
10 weeks	Clinical follow-up	Gradual resolution of acute symptoms	Continued prolonged IV/topical therapy	Stable
6 months	Recurrence	MRI: bilateral sphenoid necrosis, soft tissue enhancement into pterygopalatine fossa	Aggressive medical therapy, reduced immunosuppression	No further surgical intervention
8 months	Continued combined IV, oral, and topical antibiotics	Gradual improvement in clinical exam	Long-term antimicrobial management	Infection controlled
1 year after stopping antibiotics	Follow-up endoscopy + MRI	No recurrence	No further intervention	Patient asymptomatic, vision intact, persistent cranial nerve V2 paresthesia

## Discussion

To our knowledge, this is the first reported case of necrotizing pseudomonal sinusitis in a solid organ transplant patient. While isolated cases of pseudomonal sinonasal infections have been described, none have specifically documented the invasive necrotizing phenotype in this high-risk population. Our case highlights the need to broaden the differential diagnosis of invasive fungal-like sinusitis syndromes in transplant patients to include *Pseudomonas*, as timely recognition and targeted therapy are imperative.

The clinical findings initially appeared consistent with IFS - a feared diagnosis that presents solely in the immunocompromised population. Physicians must be aware that both forms of sinusitis have the identical clinical manifestation, which may include any or all of the following: sinus opacification on CT, periantral edema, cranial nerve palsies, vision loss, palate necrosis, and necrotic nasal mucosa on endoscopy. However, treatment for invasive pseudomonal disease may differ from that of invasive fungal disease in one respect - pseudomonal disease is more effectively treated by medical management, as suggested by previous case reports [4,5]. The largest case series of invasive pseudomonal disease by Kuan et al. suggests that conservative sinus debridement is sufficient for source control [4]. In their series, complete resolution of infection took anywhere from two to 24 months. Four out of their six patients presented with cranial nerve palsies, and three of these palsies eventually recovered. Despite the presence of a V2 neuropathy in our patient, as well as periorbital and dural inflammation on imaging, surgical debridement was limited to the sinonasal space. This was followed by antimicrobial therapy, along with modulation of his immunosuppressive regimen in close collaboration with his Transplant and Infectious Disease team. The preferred treatment for *P. aeruginosa* infections is systemic monotherapy with an anti-*Pseudomonas* β-lactam, given proven susceptibility. In contrast, dual or combination systemic therapy, such as β-lactam with an aminoglycoside, does not improve clinical outcomes and is recommended against by the Infectious Disease Society of America [7-10]. Furthermore, systemic dual therapy also has a higher risk for toxicity. The recommended duration of either intravenous or oral anti-pseudomonal therapies should be tailored to the individual patient based on clinical response, immunocompromised status, and source control [7-10]. Generally, patients are treated for approximately seven days regardless of medication route but shorter courses of therapy are preferred if possible [7-10]. In immunocompromised patients, such as the presented case, close monitoring is required, and extended systemic therapy duration is common. 

Tobramycin irrigation solution was used in this case as an adjunct treatment to decrease bacterial burden and improve symptoms. Tobramycin, an aminoglycoside antibiotic, inhibits bacterial protein synthesis and is FDA-approved for serious* P. aeruginosa *infections [11,12]. A topical delivery of this drug allows for high local drug levels required to overcome biofilm resistance while limiting toxicity, as seen in immunocompromised cystic fibrosis patients [11,12]. Its use is limited, however, in settings of previously established biofilm because of bacterial resistance. As seen in Kuan et al.'s case series, anti-pseudomonal treatment for our patient was also required long-term, ultimately being discontinued one year later [4]. Conversely, in IFS, aggressive up-front surgical clearance of disease is often performed and may include orbital exenteration, maxillectomy, or debridement within the pterygopalatine fossa. This highlights the importance of establishing a histological diagnosis prior to considering the extent of surgical treatment. Initial biopsies are often taken within the OR, and these should be carefully evaluated by frozen pathology section prior to considering the extent of debridement. In one previous report of necrotizing pseudomonal sinusitis, frozen pathology showed apparent fungal hyphae, but was later confirmed as fibrin strands on later staining, highlighting the importance of careful pathologic analysis [5].

Several factors contribute to the high virulence of *P. aeruginosa*. For instance, it secretes cytotoxins, elastase, and alkaline protease, which all directly destroy tissue, damage the extracellular matrix, and impair immune cell function [13-18]. Furthermore, *P. aeruginosa* also facilitates the formation of robust biofilms, which challenge host immune clearance and may lead to resistance to antibiotics, both of which are especially seen in immunocompromised patients [13,16]. Necrotizing pseudomonal infections are more common elsewhere in the body aside from the sinuses. Ecthyma gangrenosum (EG) is one such severe necrotizing infection of the skin, which can occur through direct inoculation or secondary to hematogenous dissemination in the setting of bacteremia [4]. EG occurs almost exclusively in the immunocompromised, particularly neutropenic, hosts and has been observed in the solid organ transplant population as well as other immunosuppressed individuals [5]. In nearly 75% of EG cases, the causative pathogen is found to be Pseudomonas, and initial treatment is with surgery followed by aggressive antipseudomonal therapy [4]. Immunosuppression is modulated when possible. Necrotizing infections involving the lungs and other soft tissues secondary to *Pseudomonas *species have also been reported, and treatment follows a similar pattern as above. It is important for the clinician to be aware of prior cultures and infectious disease work-up in the days and weeks leading up to the invasive sinusitis presentation, as these may be crucial clinical elements in identifying *P. aeruginosa* as the rare causative organism for a necrotizing sinusitis. Our patient developed a pseudomonal bacteremia two weeks prior to his presentation with sinonasal symptoms, offering a clue to his ultimate diagnosis. 

## Conclusions

Necrotizing pseudomonal sinusitis is an underrecognized syndrome, but similar to IFS and EG, has a predilection for immunocompromised hosts. Although this disease is highly morbid, it is treatable with a balance of medical and surgical management. Our case supports prior reports suggesting that surgical management may often be limited to the sinonasal cavities, with extended courses of antipseudomonal therapy being essential for favorable outcomes. However, since the current literature is limited to a handful of case reports, including ours, broader generalizations about optimal management should be made with caution. The rarity of this condition, its heterogeneous presentation, and the absence of long-term outcome data represent limitations. Nonetheless, this report underscores the importance of including bacterial etiologies, particularly *P. aeruginosa*, in the differential diagnosis of necrotizing sinusitis, as timely recognition and pathogen-directed therapy can be lifesaving, despite the risk of long-term morbidities such as paresthesia.
